# Study Protocol for the Preschooler Regulation of Emotional Stress (PRES) Procedure

**DOI:** 10.3389/fpsyg.2017.01653

**Published:** 2017-09-22

**Authors:** Livio Provenzi, Rafaela G. M. Cassiano, Giunia Scotto di Minico, Maria B. M. Linhares, Rosario Montirosso

**Affiliations:** ^1^0-3 Center for the at-Risk Infant, Scientific Institute IRCCS Eugenio Medea Bosisio Parini, Italy; ^2^Department of Neurosciences and Behavior, Ribeirão Preto Medical School, University of São Paulo Ribeirão Preto, Brazil

**Keywords:** emotion regulation, observational methods, preschoolers, stress response, study protocol

## Abstract

**Background:** Emotional stress regulation (ESR) rapidly develops during the first months of age and includes different behavioral strategies which largely contribute to children’s behavioral and emotional adjustment later in life. The assessment of ESR during the first years of life is critical to identify preschool children who are at developmental risk. Although ESR is generally included in larger temperament batteries [e.g., the Laboratory Temperament Assessment Battery (Lab-TAB)], there is no standardized observational procedure to specifically assess and measure ESR in preschool aged children.

**Aim:** Here, we describe the development of an observational procedure to assess ESR in preschool aged children [i.e., the Preschooler Regulation of Emotional Stress (PRES) Procedure] and the related coding system.

**Methods:** Four Lab-TAB emotional stress episodes (i.e., the Stranger, the Perfect Circle, the Missing Sticker, and the Transparent Box) have been selected. Independent coders developed a list of ESR codes resulting in two general indexes (i.e., active engagement and stress level) and five specific indexes (i.e., anger, control, fear, inhibition, sadness). Finally, specific actions have been planned to assess the validity and the coding system reliability of PRES procedure.

**Ethics and Dissemination:** The study has been approved by the Ethical Committee of the Scientific Institute IRCCS Eugenio Medea, Bosisio Parini (Italy). The PRES validation and reliability assessment as well as its use with healthy and at-risk populations of preschool children will be object of future scientific publications and international conference presentations.

## Introduction

### Background

The ability to cope with emotional stress [i.e., emotional stress regulation (ESR)] develops early during the first months of life (Thompson, 1994; DiCorcia and Tronick, 2011) and is a key component of infants and children’s temperament (Rothbart et al., 2011). The early development of adequate behavioral strategies of ESR is thought to be one of the major predictive factor of emotional (Eisenberg et al., 2010; Berking and Wupperman, 2012), cognitive (Compas et al., 2014; Blankson et al., 2017), and social (Cisler and Olatunji, 2012; Pisani et al., 2013) adjustment later in life. For this reason, the availability of an observational tool to assess ESR in preschool children is of crucial importance.

The Laboratory Temperament Assessment Battery (Lab-TAB; Goldsmith et al., 1993) is a set of experimental tasks specifically developed to measure preschool children’s temperament. Although it includes specific episodes which depict different ESR behavioral strategies, there is no standardized Lab-TAB procedure which allows to assess ESR in preschoolers thoroughly. In the present contribution, we present the study protocol for the development of a specific laboratory procedure to assess ESR in preschool aged children (namely, the Preschooler Regulation of Emotional Stress, PRES procedure) which is built on a comprehensive and standardized selection of specific Lab-TAB episodes which highlight behavioral strategies used to cope with emotional stress.

### ESR Conceptualization

#### Relevance of ESR for Child Development

Human infants develop adaptive behavioral strategies to cope with emotional stressors during the first months of life and within daily interactions with their main caregiver (DiCorcia and Tronick, 2011). As mother–infant and mother–child interactions are inherently characterized by frequent ruptures and reparations (Weinberg and Tronick, 1998; Beeghly et al., 2011), emotional stress is a part of infants’ interactions with their everyday human environment. Infants are thought to develop adaptive behavioral strategies to regulate emotional stress through repeated experiences of co-regulation of interactive and communicative ruptures, as they move from the need of external sources of regulation provided by the caregiver to adequate self-regulation abilities (Beeghly and Tronick, 2011; Bornstein and Manian, 2013).

Less-than-optimal strategies of ESR have been described in different subjects at-risk due to both environmental and genetic factors, including prematurity (Hsu and Jeng, 2008; Montirosso et al., 2010; Langerock et al., 2013), trauma-exposed children (Dvir et al., 2014; Szentágotai-Tãtar and Miu, 2016), and infants with specific genotypes associated with stress susceptibility (e.g., the serotonin transporter polymorphism, 5-HTTLPR; Pauli-Pott et al., 2009; Montirosso et al., 2015b). Moreover, altered ESR during the early stages of development is considered to be a critical risk factor for further behavioral and affective development during adulthood. Indeed, children characterized by emotional stress dysregulation have a heightened risk of developing psychopathology during adolescence (McLaughlin et al., 2011) and adult-age (Compas et al., 2014; Huh et al., 2017).

In the light of this evidence, standardized and well-validated approaches to ESR assessment during infancy and preschool age appear to be crucial in order to better understand the consequences of early adversities on human emotional development and to adequately plan effective preventive and therapeutic interventions.

#### Toward a Multi-faceted and Processual Conceptualization of ESR

ESR is a multifaceted construct (Aldao et al., 2016) which includes expressions and behaviors which allow individuals to cope with emotional stressors (**Table 1**). The display of emotional distress, such as negative emotionality, has been reported in infants (Braungart-Rieker et al., 1998), and specific emotional expressions, such as anger (Gilliom et al., 2002), fear (Buss and Kiel, 2011), and sadness (Compas et al., 2014) have been observed in preschool aged children. Gaze aversion with the source of emotional stress is another strategy used to regulate behavioral states during challenging conditions (Beebe and Steele, 2013). Furthermore, children might try to obtain external support from the adult caregiver (i.e., co-regulation) by making active attempts to obtain social engagement behaviors from the mother (Ekas et al., 2013; Provenzi et al., 2015a) as well as signals of protest (Ahnert et al., 2004; Kaitz et al., 2010). Finally, older infants and preschool aged children develop behavioral strategies aimed to achieve behavioral and emotional regulation (Planalp and Braungart-Rieker, 2015) which include attempts to obtain control over the situation as well as inhibition of the emotional reaction (Adrian et al., 2011).

**Table 1:** Conceptualization of ESR in infants and preschool children, with examples of the relative expressive and coping behaviors.

		Expressive behaviors	Coping behaviors
Infants	Reactivity	*e.g., Negative emotionality*	*e.g., Avoiding behaviors*
	Recovery	*e.g., Positive emotionality*	*e.g., Social engagement*
Children	Reactivity	*e.g., Negative emotions (anger, fear, sadness)*	*e.g., Inhibitory control*
	Recovery	*e.g., Positive emotionality*	*e.g., Active and social engagement*


Moreover, ESR is thought to be a two-step process that includes: a reactivity phase, during which the individual gathers strength face an external source of emotional stress and to activate specific behavioral outputs to respond and cope with the environmental challenge; and a recovery phase, during which the organism reaches a new homeostatic and quiet state when the emotional stress condition is over (Linden et al., 1997; Tsigos et al., 2000). In other words, adaptive ESR includes the adoption of adequate strategies to react to an external source of emotional stress (i.e., reactivity) as well as the return to baseline behavioral states when the stress is over (i.e., recovery).

### Available Tools to Assess ESR in Infants and Children

#### ESR Assessment during Infancy

The assessment of ESR in infants has been carried out according to different observational paradigms, including frustration tasks (Buss and Goldsmith, 1998), emotion-inducing tasks (Malone et al., 1985) and structured mother–infant interactions (Feldman, 2007). Nonetheless, the Face-to-Face Still-Face (FFSF) procedure (Tronick et al., 1978) is the most used and validated procedure to obtain information on expressive and coping behaviors adopted by infants to face emotional stress during the first months of life (Mesman et al., 2009; Provenzi et al., 2016).

During the FFSF procedure, emotional stress arises from the experimental manipulation of maternal responsiveness and availability in the interaction with the infant (i.e., maternal still-face). First, after 2 min of normal face-to-face interaction (i.e., *Play* episode), mothers are instructed to interrupt any communication with the infant, to avoid physical contact and to maintain a still/poker-face while looking their infant in the eye (Tronick et al., 1978). During this FFSF episode (i.e., *Still-Face* episode) infants are expected to exhibit specific reactivity behaviors in response to emotional stress (i.e., maternal unresponsiveness) including heightened negative emotionality and avoidant behaviors as well as reduced engagement (Adamson and Frick, 2003; Montirosso et al., 2015a). After the *Still-Face*, mothers and infants resume normal face-to-face interaction (i.e., *Reunion* episode) as during the *Play* episode. The *Reunion* episode allows the observers to obtain information about infants’ capacity to recover from emotional stress as the social engagement resumes, and the reduction of negative emotionality, despite previous research has documented that a typical carry-over effect of negative emotionality is generally observed (Yato et al., 2008; Mesman et al., 2009). As such, the FFSF procedure allows to observe infants’ behavior during both the reactivity and recovery phases of ESR.

Specific coding systems have been developed and validated for the FFSF procedure [e.g., the Infant Regulatory Score System (IRSS); the Infant-Caregiver Engagement Phases (ICEP)]. These coding systems include specific indexes of ESR behavioral strategies such as infants’ gaze direction, vocalizations, gestures, self-comforting behaviors, distancing behaviors, and general indexes of motor activity which can be resumed as expressive (i.e., negative and positive emotionality) as well as coping (i.e., social and object engagement) behavioral indexes. As such, the FFSF is also characterized by a comprehensive assessment of infants’ ESR behavioral strategies.

#### Assessment of ESR in Preschool Children

Previously adopted procedures have been developed to assess different aspects of children’s behavioral and emotional development. For example, the Strange Situation procedure has been applied to older infants and preschool aged children, but it has been developed to assess attachment-related behaviors. As such, despite the fact that ESR and attachment have known interconnections (Zimmer-Gembeck et al., 2015), the Strange Situation procedure might lack the adequate fine-grained sensitivity to depict different ESR behavioral strategies and it usually does not provide information on the two-step reactivity-recovery process. Other laboratory procedures, such as the frustration task (Melnick and Hinshaw, 2000), non-standardized stranger approaches (Zimmermann and Stansbury, 2004), rigged peer competition (Hughes et al., 2002), fear-inducing paradigms (Buss and Goldsmith, 1998), and frustration-inducing tasks (Stifter and Braungart, 1995; Cole et al., 2003) appear to be stand-alone tasks which only partially cover the different types of emotional stress that preschool aged children might face.

Notably, many of these individual tasks have been included in the Lab-TAB, which is a set of laboratory tasks specifically developed to measure temperament in preschool aged children. As ESR contributes to the definition of a temperamental profile of children (Rothbart et al., 2006), it is not surprising that the Lab-TAB includes specific episodes aimed at observing and assessing behavioral strategies used by preschool aged children to cope with emotional stress. Nonetheless, it should be noted that temperament represents a global account of children’s behavioral trait predispositions which only partially overlap with ESR (Fox and Calkins, 2003). Indeed, whereas temperament represents an overall behavioral tendency of children with innate biological underpinnings (Goldsmith et al., 1987; Rothbart et al., 2001; Saudino and Micalizzi, 2015), ESR appears to be much more dynamic and processual, contingent to environmental conditions (i.e., emotional stressors) and affected both by genetic predispositions (Lesch, 2011; Waider et al., 2011; Ford et al., 2014) and the quality of early caregiving environment (Bariola et al., 2011; Morris et al., 2011; Kim and Kochanska, 2017).

#### Limitations of the Lab-TAB to Assess Preschool Children ESR

The Lab-TAB presents a series of challenges when it comes to its application on the observation of ESR in preschool aged children. First, the Lab-TAB is not entirely specific to ESR. Although it includes emotion-eliciting episodes (Gagne et al., 2011), the available coding system is meant to provide measures of temperament (e.g., levels of activity, approach, persistence), rather than contextual regulation of emotional stress behavioral strategies. Second, the Lab-TAB is made up of more than 30 episodes (Gagne et al., 2011). Consistently, sometimes the Lab-TAB is administered in two or more sessions, and the actual duration varies according to the segmentations of the Lab-TAB procedure and to children’s characteristics (e.g., age). As such, previous researchers have selected different sets of Lab-TAB episodes in their studies. For example, 20 Lab-TAB episodes have been used with 4.5-year-old children (Gagne et al., 2011), 12 episodes at with 3 year olds, nine episodes with 6 year olds (Dyson et al., 2015), and two episodes with 12-month-old infants (Zmyj et al., 2017). Third, there is no clear available rationale to guide the selection of Lab-TAB episodes, which has resulted in the proliferating of various subjective “sub-versions” of the Lab-TAB procedure. For example, effortful control has been measured using four episodes (Car Seat, Puppets, Masks, and Risk room; Kochanska and Knaack, 2003) in 14-to-22-month-old preschoolers, behavioral inhibition has been assessed using three episodes (Dinky toys, Snack delay, and Gift; Gartstein and Marmion, 2008) in 2-year-old children, positive emotionality has been coded according to five different episodes, including Puppets, Peek-a-Boo game, Pop-up bunny, Snake, Bubbles (Kochanska et al., 2007). Finally, despite the authors of the original Lab-TAB manual provided a general guide to code children’s temperament, researchers that used the Lab-TAB have developed different coding systems depending on the objectives of their research projects, which has resulted in the production of indexes that are only partially comparable, e.g., interest, initiative, sociability, compliance (Dyson et al., 2015); anger, fear, shyness, approach, persistence (Gagne et al., 2011); negative reactivity (Shanahan et al., 2008); and two global indexes of both negative and positive emotionality (Kopala-Sibley et al., 2017).

As such, although the Lab-TAB is a well-established procedure used to assess preschool aged children’s temperament in a laboratory setting, a standardized protocol to guide its administration to evaluate preschoolers’ behavioral strategies to cope with emotional stress is non-existent up to date.

### The Present Study

In the present manuscript, we describe the PRES protocol and we provide details on (1) the theoretical and methodological reasons that guided us in choosing specific Lab-TAB stress-related episodes; (2) the procedural steps for the PRES development; (3) the operational definitions of the PRES codes and indexes; (4) the methodological steps planned to assess the coding system’s validity and reliability.

## Materials and Equipment

### Development of the Observational Procedure

#### Selection of Lab-TAB Episodes: Rationale and Description

**FIGURE 1:**
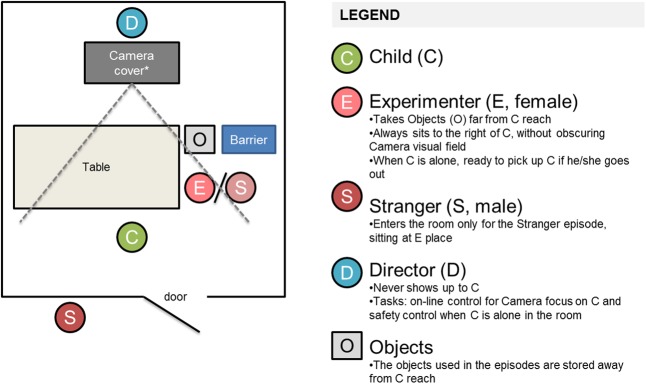
Schematic representation of the CASES setting. ^∗^The director (D) sits behind a hidden camera. The hidden camera may be behind a cloth or in a room with a unidirectional mirror next to the PRES room. The *barrier* might be a chair or a little bench, in order to avoid that the child gets to the opposite side of the table and sees the director and the camera.

The setting for the PRES procedure is graphically schematized in **Figure 1**. An essential thesaurus of PRES-related terms is provided in **Table 2**. The PRES procedure includes four episodes (i.e., Stranger, Perfect circle, Sticker, Transparent box) which have been extracted from the original Lab-TAB. These episodes have been chosen in order to represent the different types of emotional stress which have already been targeted in previous research (Gunnar et al., 2009; Adrian et al., 2011). Moreover, they have been selected in order to guarantee an easy-to-reproduce observational setting so that the procedure can be replicated in different laboratories without the need of expensive or *ad hoc* materials. The procedural steps of the four episodes are described in detail in **Table 3**.

**Table 2:** Thesaurus of PRES Procedure-related terms.

Term	Description
Session	Each child participates in a session, which is a complete PRES procedure including four episodes (i.e., Stranger, Perfect circle, Stickers, Transparent box).
Episodes	The episodes are four stress-inducing experimental tasks: Stranger, Perfect circle, Stickers, Transparent box.
Segments	Each episode is subdivided in three or more segments. The number of segments varies among episodes: Stranger (five segments), Perfect circle (three segments), Stickers (six segments), Transparent box (three segments).
Phases	For each episode, the segments are grouped into three phases: baseline, reactivity, and recovery.
Epochs	The coding procedure is micro-analytical and it is done every 10-s intervals.


**Table 3:** Description of the four PRES Procedure episodes.

#	Name	Brief description	Phases (segments)
1	Stranger approach	A male stranger gradually approaches the child physically and verbally. The stranger asks questions regarding the Child (e.g., “Have you been here already?,” “What’s your favorite toy?”), and asks about a female colleague (i.e., the Experimenter) that he is looking for. The episode ends with the Stranger saying “Hello!” to both the Child and Experimenter.	Baseline
			(segment 1: Child + Experimenter, briefing)
			Reactivity
			(segment 2: Child alone)
			(segment 3: Child + Stranger, approach)
			(segment 4: Child alone)
			Recovery
			(segment 5: Child + Experimenter, “hello!”)
2	Perfect circle	The Experimenter asks the Child to draw a perfect circle, without giving guidelines about how a perfect circle should be. For about 3 min the Experimenter says “No, this is not perfect. Try again” to every attempt made by the Child. The episode ends with the Child making a happy face out of the last “perfect” circle.	Baseline
			(segment 1: Child + Experimenter, briefing)
			Reactivity
			(segment 2: Child + Experimenter, drawing)
			Recovery
			(segment 3: Child + Experimenter, happy face)
3	No sticker left	The Experimenter asks the Child to choose one among different cartoon-related stickers. Once the Child chooses a sticker, the Experimenter says: “Ok, now wait here, I need to give the other stickers to other children around here. I’ll be back with yours in a while.” When the Experimenter comes back, she has no stickers left for the Child. The episode ends with the Experimenter finding the Child’s sticker and giving it to him.	Baseline
			(segment 1: Child + Experimenter, choosing)
			(segment 2: Child + Experimenter, describing)
			Reactivity
			(segment 3: Child alone)
			(segment 4: Child + Experimenter, no sticker)
			(segment 5: Child alone)
			Recovery
			(segment 6: Child + Experimenter, sticker back)
4	Transparent box	The Child is given a transparent box which contains an attractive toy. However, the box is closed with a lock and the Child is given the wrong key to open it. After 4 min of attempts alone, the Experimenter comes back into the room with the right key and the Child can play with the toy.	Baseline
			(segment 1: Child + Experimenter, briefing)
			Reactivity
			(segment 2: Child alone, attempts)
			Recovery
			(segment 3: Child + Experimenter, playing)


#### Emotional Stressors

The Stranger episode elicits stress due to the encounter with an unfamiliar adult that is approaching and talking to the child and with whom the child had no previous relational or interactive history. As such, this episode is meant to elicit fear-related stress.

The Perfect circle episode elicits stress due to frustration and perception of self-inefficacy within a relational framework with an adult experimenter who gives negative feedbacks about the graphical production of a circle and asks for further drawing attempts without providing guidelines on how a “real” perfect circle should be drawn.

The Sticker episode induces stress due to the mismatch between the expectation of a reward (i.e., the chosen sticker) and the lack of the desired adult response (i.e., absence of the chosen sticker). As such the Perfect circle and the Sticker episodes both elicit frustration-related stress in the child, but, whilst the first is caused by a perception of self-inadequacy (e.g., being unable to draw a perfect circle), the second is triggered by an attribution of inadequacy to the other (e.g., being unable to maintain a promise).

Finally, the Transparent box episode elicits stress due to the simultaneous presence of a visible desirable object within a transparent container, and the impossibility to reach it. As such, this task is meant to observe the perseverance – or lack thereof – of the child’s efforts to achieve a desired goal while facing an impossible task. Moreover, the emotional stress elicited in this fourth episode is related to the presence of a desirable object within sight which cannot be reached nor played with by the child.

## Stepwise Procedures

### Development of the Micro-analytical Coding System

#### Naïve Coding of Infant and Child Observation

A first set of unstructured and non-hierarchical codes was developed by two researchers (authors LP and RMC), who hold expertise in the coding of infants and children’s behavior. Each coder independently watched 10 pilot applications of the PRES and provided a list of potential codes of the child’s behavior every 10 s. Coders were asked to annotate the exact timing at which the selected behavioral codes occurred in order to facilitate the subsequent consensus discussion. In order to allow the coders to produce an adequate number of potential codes, no theoretical nor methodological limitations were imposed and descriptive rather than conceptual language was encouraged.

#### Consensus Procedure for Univocal Coding System

Subsequently, the first set of codes underwent a consensus discussion between the researchers. Specifically, overlapping codes identified by both LP and RMC survived the first screening, whereas codes present in the list of only one coder were discussed in *ad hoc* meetings. When a code was proposed only by one coder, different scenarios were possible. First, coders checked for potential overlapping of the code with previously identified and accepted codes. Second, if not overlapping, the time-frame in which the code occurred was reviewed by both coders together with a senior researcher (author RM). After this consensus process, the code was either suppressed or included. The final set of selected codes is reported in **Table 4**. They were separated in general codes (**Table 4A**) and specific codes (**Table 4B**), on the basis of their occurrence throughout the entire PRES procedure or limitedly to specific episodes, respectively.

**Table 4:** List of general **(A)** and specific **(B)** codes.

(A)		

Codes	Levels	Description
Activity level	Sit	The child sits on the chair
	Stand	The child stands up in front of the table
	Exploring	The child is exploring the environment, moving around the room
Emotional state	Positive	Facial, body or vocal expressions of positive affect
	Neutral	Absence of clear signs of positive or negative affect
	Negative	Facial, body or vocal expressions of negative affect
Gaze direction	Object-directed	The child maintains eye-contact with a task-related object
	Adult-directed	The child maintains eye-contact with a task-related adult
	Gaze aversion	The child averts eye-contact with task-related objects/adults
Peripheral movements	Hands	Subtle hands movements (e.g., grasping, rubbing, playing nervously) which are not associated with communications, playing, task-related activities or exploring
	Mouth	Subtle mouth movements (e.g., grimaces, biting the lips, tongue movements, stretching the lips) which are not associated with communications, playing, task-related activities or exploring
	Legs	Leg movements which are not embedded in general movements of the child (e.g., not related to postural changes)
	Arms	Arm movements which are not embedded in general movements of the child (e.g., not related to postural changes)

**(B)**		

**Episodes**	**Codes**	**Specific index**

1	Child waits for the experimenter when she exits	Control
	Child moves toward the door when the experimenter is out of the room	Control
	Child asks about the experimenter where she is going when she exits	Control
	Child asks where the experimenter is to the stranger	Control
	Child checks for the experimenter out of the door	Control
	Child moves away from the stranger	Fear
	Child opens the door to exit	Fear
	Child does not respond to the stranger’s questions	Inhibition
	Child does not describe the stranger to the experimenter	Inhibition
	Child needs help to describe the stranger to the experimenter	Inhibition
	Child has no memory of the stranger	Inhibition
2	Child expresses anger or protest (verbal and non-verbal)	Anger
	Child asks how the perfect circle should be drawn	Control
	Child looks at the experimenter instead of drawing	Control
	Child looks at the experimenter waiting to receive a feedback	Control
	Child asks to turn the sheet	Control
	Child does not draw the circle	Inhibition
	Child does not draw the smiling face	Inhibition
	Child expresses sadness or resignation	Sadness
3	Child expresses anger or protest (verbal and non-verbal)	Anger
	Child asks about the experimenter where she is going when she exits	Control
	Child waits for the experimenter when she exits	Control
	Child opens the door to exit	Fear
	Child asks about stickers’ destiny	Control
	Child does not choose any sticker	Inhibition
	Child does not describe the chosen sticker	Inhibition
	Child does not accept back the sticker	Inhibition
	Child expresses sadness or resignation (verbal and non-verbal)	Sadness
4	Child expresses anger or protest (verbal and non-verbal)	Anger
	Child asks about the experimenter where she is going when she exits	Control
	Child opens the door to exit	Fear
	Child gives up at attempting to open the box	Inhibition
	Child expresses sadness or resignation (verbal and non-verbal)	Sadness
	Child does not play with the toy	Inhibition
	Child plays with the toy in aggressive ways	Anger


#### Computing of the ESR General and Specific Indexes

The coding of the PRES procedure is micro-analytical (i.e., 10-s epochs). Every 10 s, the coders have to attribute a level of each general and specific code. A series of algorithms was developed in order to obtain general and specific indexes starting from general and specific codes, respectively. Prior to the computation of general and specific indexes of ESR, each code was weighted on the actual duration (i.e., number of epochs) of each episode’s phase (i.e., baseline, reactivity, and recovery). As such, every code is expressed as a proportion ranging from 0 (never occurring) to 1 (always occurring).

*General indexes.* General codes are resumed into two general indexes: active engagement and stress level. General indexes are computed separately for the baseline, reactivity and recovery phases of each PRES episode.

*Active engagement* is calculated as the sum of the following general codes: sit (activity level), positive and neutral (emotional state), adult-directed and object-directed gaze (gaze direction). As such, active engagement ranges from 0 (all the included codes never occur throughout the specific phase of the episode) to 4 (all the included codes occur during every epoch of the specific phase of the episode).

*Stress level* is computed as the sum of the following general codes: stand (activity level), negative (emotional state), hands and mouth movements (peripheral movements), gaze aversion (gaze direction). As such, stress level ranges from 0 (all the included codes never occur throughout the specific phase of the episode) to 4 (all the include codes occur during every epoch of the specific phase of the episode).

*Specific indexes.* The development of specific indexes of ESR followed a qualitative labeling procedure. The two independent coders attached a one-word label to each specific code. For instance, the specific code “The child moves away from the stranger” was labeled *Fear*; the specific code “No response to stranger” was labeled *Inhibition*. Furthermore, disagreement between the two coders’ labeling was discussed and resolved. Five labels were produced, corresponding to the final set of five specific indexes: *anger*, *control*, *fear*, *inhibition*, and *sadness*.

Each specific index is computed as the sum of the related specific codes (weighted on the actual duration of each episode’s phase, i.e., number of epochs). Due to the different types of emotional stress elicited by the PRES episodes, specific indexes cannot be scored for all episodes. Consistently, as varying theoretical ranges apply to different episodes, they are meant to be standardized, with mean = 0 and standard deviation = 1.

## Anticipated Results

### Proofs of Reliability

Reliability of the coding system will be assessed using multiple methods. First, inter-coder reliability will be measured according to Cohen’s *k* coefficient and percentage agreement. Second, test–retest reliability will be assessed according to Cronbach’s alpha. Finally, a confirmatory factorial analysis will be used to verify if the theoretically aggregated general and specific indexes are supported by statistic clustering.

### Strengths and Limitations

The PRES presents specific advantages and potential strengths when compared to available procedures in literature. It has been developed within a well-defined theoretical framework (i.e., infant research tradition) in which both reactivity and recovery are included as two adaptive steps of ESR. Second, the coding system is micro-analytical, which allows researchers to obtain fine-grained information on children’s behavioral responses to emotionally challenging conditions with no need of abstraction or global ratings. Moreover, the procedural and descriptive definition of each code is meant to facilitate the agreement among independent coders and to limit the risk of subjective interpretations. Third, it provides two levels of information on children ESR, including general indexes of negative emotionality and interactive engagement as well as specific indexes of different emotional responses to a stressful condition. Fourth, the procedure includes different challenging conditions which represent the main sources of emotional stress in preschool aged children. As such, the PRES is meant to be a multi-faceted and comprehensive assessment of ESR in preschoolers with a unified theoretical background, which limits the need of integrating different procedures or protocols in future research in the field.

Nonetheless, potential limitations exist. First, the PRES has been developed as a laboratory procedure. As such, its application to naturalistic settings (e.g., primary care, home environment) may require adaptations. Second, the micro-analytical nature of the PRES coding system is time- and resource-consuming. As such, despite the PRES is well-suited for research purposes, its application to clinical settings in which more rapid assessments are needed to sustain health-related decisions is limited. Third, the PRES does not include a direct evaluation of ESR in the context of peer-related stress. Despite the Stickers episode elicits stress that is related to the unequal treatment of the subject compared to non-present peers, there are no PRES episodes during which the child is required to interact with other children of the same age. Peer relationships involve many different dimensions (e.g., competition and cooperation) which are only limitedly linked with ESR and which require specific observational methods.

### Ethics and Dissemination

The study has been approved by the Ethical Committee of the Scientific Institute IRCCS Eugenio Medea, Bosisio Parini (Italy). The PRES is intended to provide micro-analytical, intensive and rich information on the socio-emotional stress regulation of preschool children and the quantitative nature of this observational procedure is suitable within cross-sectional studies comparing low- and high-risk children as well as within longitudinal studies assessing the long-term effects of early adversities on emotional development. For instance, the PRES procedure and the related coding system is currently being used in a prospective longitudinal research project on the epigenetic correlates of early adversity exposure in prematurity (the preschool-age phase is currently ongoing and data on the infancy phase are published: Provenzi et al., 2015b; Montirosso et al., 2016a,b). In this study, a clinical group of preschool aged children born preterm, which are known to be at risk of altered ESR (Montagna and Nosarti, 2016), will be compared to a control group of full-term preschool peers at 4.5 years. The comparison of the response to the PRES procedure between the two groups will also serve as a preliminary validation of the capacity of this laboratory assessment paradigm to depict difficulties in ESR in at-risk preschool aged children. Further methodological steps of the PRES validation and its application to larger samples of low- and high-risk preschool children will be reported in future conference presentations and peer-reviewed journals.

## Author Contributions

LP and RC developed the first version of the manual and the coding system. LP wrote the first draft of the present manuscript. GSdM contributed to final manuscript editing and English editing. ML and RM approved the final version of the manuscript. RM contributed to the refining of the manual, the coding system and the final paper.

## Conflict of Interest Statement

The authors declare that the research was conducted in the absence of any commercial or financial relationships that could be construed as a potential conflict of interest. The reviewer SS and handling Editor declared their shared affiliation.
